# Autism Spectrum Screening Checklist (ASSC): The Development of a Scale to Identify High-Risk Individuals Within the Children's Mental Health System

**DOI:** 10.3389/fpsyt.2021.709491

**Published:** 2021-09-06

**Authors:** Shannon L. Stewart, Angela Celebre, Jo Ann Iantosca, Jeffrey W. Poss

**Affiliations:** ^1^Faculty of Education, Western University, London, ON, Canada; ^2^Faculty of Applied Arts and Health Sciences, Seneca College, Toronto, ON, Canada; ^3^Faculty of Applied Health Sciences, University of Waterloo, Waterloo, ON, Canada

**Keywords:** interRAI, children and youth, mental health, autism spectrum disorder, screening

## Abstract

Autism Spectrum Disorder (ASD) is a complex childhood onset neurodevelopmental disorder that has become the fastest growing developmental disability. Due to the increased demand for diagnostic assessments and subsequent increased wait times, standardized screening as part of regular clinical practice is needed. More specifically, there is an important need for the development of a more streamlined screening tool within an existing assessment system to identify those at greatest risk of having ASD. The current study utilized data from ~17,000 assessments obtained within the province of Ontario, based on the interRAI Child and Youth Mental Health (ChYMH) and Child and Youth Mental Health and Developmental Disability (ChYMH-DD), to develop a scale to identify children who have a higher likelihood of having autism. The scale was then tested on a trial population with data from the interRAI Early Years instrument. Further analyses examined the predictive validity of the scale. The Autism Spectrum Screening Checklist (ASSC) was found to be a good predictor of ASD with a sensitivity of 0.73 and specificity of 0.62, at the recommended cut-point of 2+. The results were consistent across several age ranges, specifically from 2 to 21 years of age. The ASSC scale provides an initial screen to help identify children and youth at heightened risk for autism within larger populations being assessed as part of routine practice. The main goal for the development and implementation of the ASSC scale is to harness the power of the existing interRAI assessment system to provide a more efficient, effective screening and referral process. This will ultimately help improve patient outcomes through needs-based care.

## Introduction

Autism spectrum disorder (ASD) is a complex, lifelong, neurodevelopmental disorder that is characterized by impairment in social communication and the presence of restricted repetitive behaviors (1, 2). ASD has a variety of causes, such as those at the genetic, biological, and environmental level (3). Some of the earliest signs reported by parents include lower levels of social communication and attention, increased repetitive behavior, and temperament dysregulation (4). However, there is also substantial heterogeneity in its presentation and overlap with other developmental disorders, especially in the first few years of life, thereby adding to the complexity of the diagnostic process (5, 6). Autism is also highly co-morbid with intellectual disability, with estimates around 50–70% being reported in the literature (7). Notably, ASD is associated with substantial disability across the lifespan, which is only exacerbated when interventions are not provided early on (8, 9).

Autism is one of the most common childhood onset neurodevelopmental disorders. It is estimated that the current prevalence rate is ~1–1.5% of the world's population (10). With respect to the pediatric population in particular, recent estimates indicate that 1 in 68 school-aged children has been identified with ASD in the United States (11). Autism has also been deemed the fastest growing developmental disability, with a steady increase in reported prevalence over the past decade (12). Increased prevalence rates have led to increased demand for diagnostic assessments, which typically exceeds available resources and results in increased wait times. Importantly, this waiting time occurs during a critical period of brain development, and so lengthy wait times may delay intervention and decrease its effectiveness (8, 13). Indeed, there is substantive research showing that early intervention is key to achieving better prognostic outcomes (14, 15). This waiting period also represents a highly stressful time for families (16, 17). As such, studies have found that many parents are frustrated and dissatisfied with the diagnostic process and experience it as slow, stressful, and poorly managed (18, 19). Three recent ASD guidelines have recommended a maximal wait time of 3 to 6 months, yet the average wait time between parents' first concerns around their child's developmental progress and diagnosis is 2 to 4 years (20). This discrepancy can help explain the consistent finding that the average age of diagnosis is 4–5 years-old despite the fact that ASD can be reliably detected within the second year of life (20).

Various factors account for the more than 2 year difference between parents noticing the early signs of autism to receiving a diagnosis. Some of these barriers include time-consuming evaluations as well as a lack of providers who are able to administer the diagnostic assessments (21, 22). Other important barriers include inappropriate referrals (or more specifically, over-referrals), and a lack of effective screening tools (23–25). In a comprehensive review of early autism screening, the authors concluded that their findings emphasize the need for “a more efficient, intelligent, and innovative ASD screening tool” (p. 24) (25). Some instruments: (1) are time-consuming to administer, (2) have an unacceptable level of sensitivity (e.g., 40%), and (3) are not comprehensive in terms of the population served; for example, some of the screening tools were only intended to be used on infants from 16 to 36 months, whereas others were strictly meant for adolescents/adults.

Finally, an overarching barrier to early identification and diagnosis is ineffective care pathways (23). Improved care pathways are needed to reduce waiting times for an ASD diagnostic assessment and direct each child to more appropriate services. More explicitly, there is a critical need for the development of a more streamlined, easily implemented, resource-effective screening method to identify those at greatest risk of having ASD and require a more comprehensive follow-up. This will help facilitate earlier diagnosis and, as a result, earlier intervention and better patient outcomes.

The aim of the present study was to develop a methodology for identifying children who are at greatest risk of having autism within the children's mental health system in the province of Ontario, Canada. Since no effective, easily implemented screening method exists, an effort was launched to develop a new scale for identifying individuals who have an increased likelihood of autism. The Autism Spectrum Screening Checklist (ASSC) was created to assist service providers in determining whether a toddler, child or adolescent is at higher risk of having ASD. This scale is embedded in an assessment-to-intervention system that is already used as standard practice across Ontario in most child and youth mental health agencies to foster effective, evidence-informed care pathways. The aim of this study is to describe the development of the ASSC scale.

## Methods

### Sample

Data came from assessments of children and youth receiving mental health services in Ontario, Canada. The derivation sample came from individuals aged 4–21 years assessed with the Child and Youth Mental Health (ChYMH) (26) or the Child and Youth Mental Health and Developmental Disability (ChYMH-DD) (27) instruments, as part of regular clinical practice from 54 agencies from 2012 to 2020. An additional sample of 2 and 3 year-old children assessed with the interRAI Early Years instrument (28) was used for a trial application, and collected from 15 agencies from 2017 to 2020. These assessment instruments are described below. Assessed individuals were referred to these agencies through a variety of sources including family and specialty physicians, school personnel, other allied health professionals, or parents/primary caregivers. Assessment information is used for a variety of purposes, including standardized care planning, as well as the use of items and calculated outcome measures to inform decision making and to track individual change.

There were 16,955 individuals in the derivation dataset, using the first assessment if an individual had more than one. The mean age was 11.95 years (SD 3.50) and 55.9% were male. There were 724 individuals in the trial application dataset of 2 and 3 year-old children, where the mean age was 2.48 (SD 0.52) and 68.8% were male. To examine predictive validity, a sub-sample of 318 individuals was used from the original derivation sample. The mean age was 11.02 years (SD 4.00) and 64.5% were male.

Assessors completed a 2.5 day training of each of the three interRAI Child and Youth instruments: ChYMH, ChYMH-DD, and the interRAI Early Years. The trained child/youth mental health professionals included psychologists, nurses, psychiatrists, speech and language therapists, child and youth workers, developmental social service workers, and social workers. All available sources of information are utilized to complete the assessment (i.e., family members, community members, document review, and clinical observations).

Secure web-based software was utilized to record assessment information, requiring responses of the proper form for all essential items before the record can be authorized as complete. Before making the data available for analysis, personal identifiers were removed. Western University's ethics board granted approval for the secondary analysis of data collected in various agencies throughout the province of Ontario (REB #106415).

### Measures

The *interRAI Early Years* is a new instrument within the interRAI child/youth suite and has been designed for young children under the age of 4 years who are referred for assessment due to mental health, relational and/or developmental concerns (28). It provides unique information tailored to early identification and intervention (e.g., prenatal complications; family and social relations; temperamental characteristics; risks related to development and mental health). It also provides a comprehensive assessment of individual needs with applications that can be used to support decisions related to care planning and outcome measurement. There are compatible items in use across care domains that share design features such as a specified observation period or time frame, a focus on observable behaviors, the use of a few, powerful questions to assess areas of need, and the use of professional judgment to integrate multiple sources of information. The interRAI Early Years is compatible with other interRAI instruments across services and sectors (e.g., mental health, education, adult sectors), relevant for all age groups across the lifespan.

The *interRAI ChYMH* and *ChYMH-DD* are comprehensive, clinician-rated, standardized, and multi-sectoral mental health instruments for children and youth (26, 27). These instruments include over 400 items and build a comprehensive picture of the child's strengths, needs, functioning, and areas of risk to inform care-planning for clients with mental health needs. The clinician creates a clinical profile of children based on a collection of reports, observations, and judgments made from interactions with the family, the children themselves, and service providers with appropriate consent. Each instrument contains evidence-based items, scales, and domains relevant to the population used in this study. While the ChYMH was designed for children 4–18 years of age with potential mental health issues, the ChYMH-DD covers a range of common issues in children with global developmental delays or intellectual disabilities from 4–21 years of age. The items are tailored to the needs of children and youth with intellectual and developmental disabilities and mental health concerns in inpatient and outpatient settings as part of standard of care. Assessors rate a child/youth on a number of demographic variables, family, mental health, and physical health indicators.

For all three instruments, clinicians receive an item-by-item interpretation guide to the interRAI instruments with information regarding intent, definition, process, and proper coding method of each item to ensure accurate and uniform assessment of children/youth across multiple mental healthcare settings. The ChYMH, ChYMH-DD and interRAI Early Years include a subsection called “*Diagnostic and other health information,”* which collects diagnostic information on 12 provisional categories, including ASD, as determined by a psychiatrist, psychologist, or attending physician.

These instruments were designed to provide a comprehensive assessment to support enhanced individualized care planning (29–32), while providing clinical decision-support algorithms (33–36) to foster evidence-based prioritization/triaging. Notably, the relatively new interRAI Early Years provides 17 care planning protocols pertinent to specific areas of need (37); for example, attachment, sleep, caregiver distress, gross and fine motor skills, and sensory issues.

Strong reliability and validity for the scales and algorithms on the interRAI ChYMH and ChYMH-DD have been found (38–42). These instruments have several applications including outcome measurement, resource allocation, and case-mix systems (34, 35, 43–46).

### Analysis

We sought to create a calculated scale that would explain a diagnosis of autism. The dependent variable was “Autism Spectrum Disorder” as a provisional diagnosis, for which the assessor records if a psychiatrist, psychologist, or attending physician has made this diagnosis. It is important to note that the assessor is not acting in any diagnostic capacity and is merely consulting all available sources of information to determine if such a diagnosis has been made. The recorded item requires the assessor to rank any of a number of provisional diagnoses by importance (most, second most, etc.); for our dependent variable, we collapsed a diagnosis of autism of any importance to be one, otherwise zero.

A list of potential explanatory variables was generated. All items in the ChYMH or ChYMH-DD instruments were considered by a clinical expert for their potential association with autism, and seven candidate items were selected (e.g., narrowly restricted range of interest and excessive preoccupation with activity or routine). Furthermore, bivariate associations between an autism diagnosis and other items in the derivation dataset were used to identify a small number of additional items that offered statistical strength. However, these additional items were not pursued, either because they were not available in the interRAI Early Years assessment instrument, or they were considered problematic for use with very young children (e.g., positive symptoms or having at least one friend). The seven candidate items were all binary. The last 3 days was the reference timeframe.

Using multivariable logistic regression, these seven items were tested together to assess their ability to independently predict an autism diagnosis and to remove non-contributing items. A series of tests using the count of retained items was then applied, with logistic regression of the count of the items to assess goodness of fit, and correlation analysis to provide a Cronbach alpha value for the individual items to inform internal consistency of the contributing items. Sensitivity and specificity for different summed scale cut-points were considered. The selected scale was tested in a trial population using the interRAI Early Years cases where it could be calculated. Furthermore, using a sample of 318 ChYMH or ChYMH-DD assessments, the ASSC scale was calculated, and longitudinal analyses related to predictive validity were conducted. Specifically, the sample included individuals for whom provisional diagnostic assignment (for all DSM-IV diagnoses, including ASD) had not been completed at the time of initial assessment, and a follow-up assessment within 365 days where it had been subsequently done. This allowed the scale to operate more like a predictive measure where a child or youth not yet subjected to diagnostic assignment at the time the scale is assigned is subsequently diagnosed, either positively or negatively, for autism.

## Results

**Figure 1:**
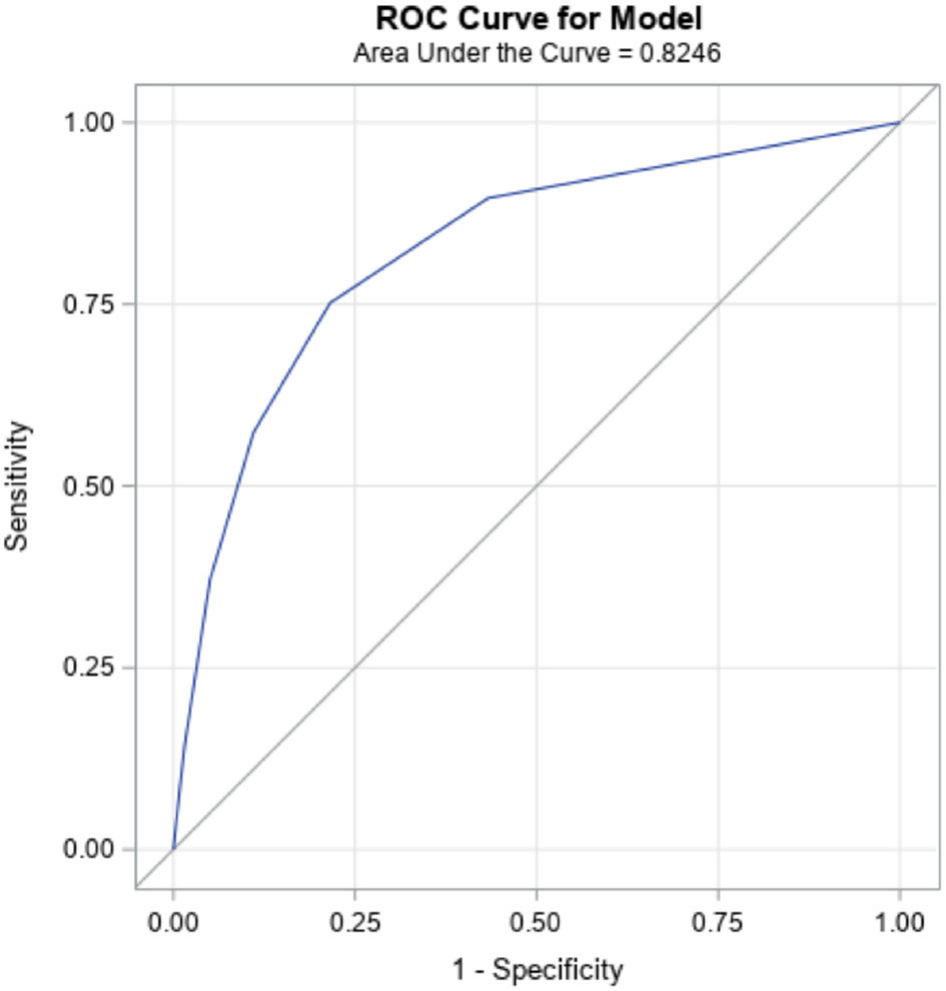
Receiver Operator Characteristics (ROC) Curve: Five-item scale.

The seven candidate items and their distribution by autism diagnosis are summarized in Table 1, along with sample characteristics. One item, “lack of interest in social interaction”, was dropped because of a weaker association and also because it was not available in this form in the interRAI Early Years instrument. Progression of the analysis is summarized in Table 2. Step 1 used the six items, giving a Cronbach alpha of 0.702 that would be increased to 0.723 if the item “self-injurious behavior” was removed. When this was done (step 2), the model fit did not decrease, making the five-item construction superior to the six-item one. As one additional variation, the weakest item of these five, “difficulty adapting to even minor change”, was dropped, resulting in a four-item sum (step 3) with a small drop in internal consistency but no change in model fit. Distribution of the summed items, odds ratios of each sum total, as well as sensitivity, specificity, positive predictive value (PPV) and negative predictive value (NPV) for sum cut-points are also provided in Table 2. Receiver-Operator Characteristics (ROC) curve plot of the five-item sum is shown in Figure 1.

**Table 1:** Sample characteristics.

***N* (%)**	**Prevalence**		
	**All**	**Autism Dx**	**No autism Dx**
N (percent of sample)	*16,955 (100%)*	*2,111 (12.5%)*	*14,844 (87.5%)*
Mean age (std)	11.9 (3.50)	11.7 (3.36)^‡^	12.0 (3.52)
Males	9,478 (55.9%)	1,595 (75.6%)^‡^	7,883 (53.1%)
Assessed as inpatient	1,145 (6.8%)	167 (7.9%)^‡^	978 (6.6%)
Assessed in person	11,472 (68.2%)	1,072 (51.5%)^‡^	10,400 (70.5%)
Walked independently by 18 months	13,308 (78.8%)	1,602 (76.7%)^‡^	11,706 (79.1%)
Talked^**^ by 24 months	11,899 (70.5%)	1,075 (51.5%)^‡^	10,824 (73.1%)
Toilet trained, daytime by 4 years	12,860 (76.2%)	1,326 (63.5%)^‡^	11,534 (77.9%)
Cognitive skills independent, makes safe decisions	9,160 (54.0%)	529 (25.1%)^‡^	8,631 (58.1%)
Communication: expresses ideas without difficulty	12,291 (72.5%)	997 (47.2%)^‡^	11,294 (76.1%)
Referral reason: threat or danger to self	5,057 (29.9%)	810 (38.8%)^‡^	4,247 (28.7%)
Referral reason: threat or danger to others	4,592 (27.2%)	955 (45.8%)^‡^	3,637 (24.6%)
**Candidate scale items**			
Self-injurious behavior	4,908 (29.0%)	937 (44.4%)^‡^	3,971 (26.8%)
Narrowly restricted range of interest	2,977 (17.6%)	1,217 (57.7%)^‡^	1,760 (11.9%)
Excessive preoccupation with activity or routine	3,330 (19.6%)	1,184 (56.1%)^‡^	2,146 (14.5%)
Lack of social/emotional conventions when socializing	3,919 (23.1%)	1,298 (61.5%)^‡^	2,621 (17.7%)
Lack of interest in social interaction^*^	5,494 (32.4%)	923 (43.7%)^‡^	4,571 (30.8%)
Excessive or unusual reaction to sensory stimuli	4,026 (23.8%)	1,139 (54.0%)^‡^	2,887 (19.5%)
Difficulty adapting to even minor change	3,770 (22.2%)	934 (44.2%)^‡^	2,836 (19.1%)

* *Item not in the interRAI 0–3 instrument*.

** *Combined 2–4 words into short sentences AND had vocabulary from 50 to 200 words*.

‡ *Autism significantly different from no-autism stratum*.

**Table 2:** Sequential steps and results: Derivation sample.

***N* = 16,955; 2,111 with autism diagnosis (12.45%)**	**Step 1: 6 items**		**Step 2: 5 items**		**Step 3: 4 items**	
**Odds ratios (95% CI), adjusted for other items used in this list of 6:**						
1. Self-injurious behavior	1.26 (1.13–1.40)		Not included		Not included	
2. Narrowly restricted range of interest	3.24 (2.84–3.69)		3.28 (2.88–3.74)		3.33 (2.92–3.79)	
3. Excessive preoccupation with activity or routine	1.86 (1.63–2.13)		1.88 (1.64–2.14)		1.97 (1.73–2.24)	
4. Lack of social/emotional conventions when socializing	2.87 (2.56–3.22)		2.90 (2.59–3.26)		2.97 (2.65–2.33)	
5. Excessive or unusual reaction to sensory stimuli	2.31 (2.07–2.57)		2.36 (2.12–2.64)		2.44 (2.19–2.71)	
6. Difficulty adapting to even minor change	1.22 (1.08–1.37)		1.25 (1.11–1.40)		Not included	
Sum of item *N* (%): 0	6,920 (40.8%)		8,636 (50.9%)		9,644 (56.9%)	
1	4,140 (24.4%)		3,529 (20.8%)		3,397 (20.0%)	
2	2,316 (13.7%)		1,936 (11.4%)		1,719 (10.1%)	
3	1,483 (8.8%)		1,324 (7.8%)		1,363 (8.0%)	
4	1,079 (6.4%)		1,001 (5.9%)		832 (4.9%)	
5	709 (4.18%)		529 (3.1%)		n/a	
6	308 (1.8%)		n/a		n/a	
Odds ratios (95% CI), sum of items: 0	Ref		Ref		Ref	
1	3.15 (2.57–3.68)		3.61 (3.02–4.31)		3.97 (3.38–4.67)	
2	7.47 (6.12–9.13)		9.19 (7.72–10.95)		11.18 (9.51–13.15)	
3	15.93 (13.05–19.45)		18.40 (15.42–21.95)		25.29 (21.54–29.71)	
4	33.01 (26.96–40.41)		35.53 (29.60–42.64)		44.28 (36.91–53.12)	
5	44.81 (35.99–55.78)		50.11 (40.30–62.31)		n/a	
6	60.58 (45.88–79.99)		n/a		n/a	
c-statistic	0.824		0.825		0.825	
Cronbach alpha	0.702		0.723		0.712	
**Sum of items predicting autism diagnosis, sensitivity and specificity with 95% confidence intervals**	**Sens**	**Spec**	**Sens**	**Spec**	**Sens**	**Spec**
1+	0.929 (0.919–0.940)	0.456 (0.448–0.464)	0.896 (0.883–0.909)	0.567 (0.559–0.575)	0.869 (0855–0.884)	0.631 (0.623–0.639)
2+	0.803 (0.786–0.819)	0.717 (0.710–0.724)	0.752 (0.733–0.770)	0.784 (0.778–0.791	0.701 (0.681–0.720)	0.836 (0.830–0.842)
3+	0.648 (0.627–0.668)	0.851 (0.845–0.857)	0.574 (0.553–0.595)	0.889 (0.884–0.894)	0.499 (0.478–0.520)	0.923 (0.919–0.927)
4+	0.465 (0.444–0.487)	0.925 (0.921–0.929)	0.370 (0.350–0.391)	0.950 (0.946–0.953)	0.223 (0.205–0.241)	0.976 (0.973–0.978)
5+	0.250 (0.232–0.269)	0.967 (0.964–0.970)	0.142 (0.127–0.157)	0.985 (0.983–0.987)	n/a	n/a
6+	0.083 (0.072–0.095)	0.991 (0.990–0.993)	n/a	n/a	n/a	n/a
**Sum of items predicting autism diagnosis, PPV and NPV with 95% confidence intervals**	**PPV**	**NPV**	**PPV**	**NPV**	**PPV**	**NPV**
1+	0.196 (0.188–0.203)	0.979 (0.975–0982)	0.227 (0.218–0.236)	0.975 (0.971–0.978)	0.251 (0.241–0.261)	0.971 (0.968–0.975)
2+	0.287 (0.276–0.299)	0.962 (0.959–0.966)	0.331 (0.318–0.345)	0.957 (0.953–0.961)	0.378 (0.363–0.393)	0.952 (0.958–0.955)
3+	0.382 (0.366–0.398)	0.944 (0.941–0.948)	0.425 (0.407–0.443)	0.936 (0.932–0.940)	0.480 (0.459–0.501)	0.928 (0.924–0.933)
4+	0.469 (0.447–0.490)	0.924 (0.920–0.928)	0.511 (0.486–0.536)	0.914 (0.909–0.918)	0.566 (0.532–0.600)	0.898 (0.894–0.903)
5+	0.519 (0.489–0.50)	0.901 (0.896–0.905)	0.567 (0.525–0.609)	0.890 (0.885–0.895)	n/a	n/a
6+	0.571 (0.516–0.627)	0.884 (0.879–0.889)	n/a	n/a	n/a	n/a

The five-item version was ultimately selected based on it having more parsimony than the six-item version, and the best internal consistency of the three options. However, all three versions are quite similar in performance with strong goodness of fit; c-statistics are >0.82, which is considered to be a strong result (47).

The five-item summative scale was applied to the interRAI Early Years assessments, where 9.5% of the cases had a diagnosis of autism; the results are summarized in Table 3. Distribution of the summed items and sensitivity, specificity, PPV, and NPV for sum cut-points are also provided in Table 3. The c-statistic is slightly higher than that in the derivation cases, and the Cronbach alpha value is slightly lower. Regarding the distribution, the interRAI Early Years cases tended to be in the lower risk categories, consistent with this group having a lower likelihood of an autism diagnosis.

**Table 3:** Five-item scale in trial population (age 3 and younger).

***N* = 724; 69 with autism diagnosis (9.53%)**	**5 item scale**			
Sum of item *N* (%): 0	454 (62.7%)			
1	138 (19.1%)			
2	67 (9.3%)			
3	46 (6.4%)			
4	14 (1.9%)			
5	5 (0.7%)			
c-statistic	0.842			
Cronbach alpha	0.646			
**Sum of items predicting autism diagnosis(95% confidence intervals)**	**Sens**	**Spec**	**PPV**	**NPV**
1+	0.884 (0.809–0.960)	0.681 (0.645–0.717)	0.226 (0.176–0.276)	0.982 (0.970–0.995)
2+	0.681 (0.571–0.791)	0.870 (0.845–0.896)	0.356 (0.274–0.438)	0.963 (0.948–0.978)
3+	0.391 (0.276–0.507)	0.942 (0.924–0.960)	0.415 (0.296–0.535)	0.936 (0.918–0.955)
4+	0.159 (0.073–0.246)	0.988 (0.979–0.996)	0.579 (0.357–0.801)	0.918 (0.897–0.938)
5+	0.044 (0.000–0.092)	0.997 (0.993–1.000)	0.600 (0.171–1.000)	0.908 (0.887–0.929)

Using a sub-sample from the derivation dataset, the predictive validity of the ASSC score was investigated. Distribution of the summed items and percentage with autism diagnosis at follow-up are provided in Table 4, along with sensitivity, specificity, PPV, and NPV for sum cut-points. In utilizing this approach, cut-points of 1+ and 2+ would provide PPVs of 29.5 and 36.7%, respectively. However, it should be noted that these higher achieved PPVs relate directly to the higher prevalence of a future autism diagnosis at 23.3%, compared to a 12.5 and 9.5% prevalence rate in the derivation sample and trial population, respectively.

**Table 4:** Longitudinal analysis: High risk for future autism diagnosis by Autism Spectrum Screening Checklist (ASSC) score at baseline.

***N* = 318; 74 with autism diagnosis at follow-up (23.3%)**	**5 item scale**		**% with autism diagnosis at follow-up**	
Sum of item *N* (%): 0	81 (25.5%)		4.9%	
1	90 (28.3%)		17.8%	
2	68 (21.4%)		25.0%	
3	35 (11.0%)		45.7%	
4	35 (11.0%)		42.9%	
5	9 (2.8%)		66.7%	
c-statistic	0.738			
Cronbach alpha	0.559			
**Sum of items predicting autism diagnosis**	**Sens**	**Spec**	**PPV**	**NPV**
1+	0.946 (0.894–0.998)	0.316 (0.257–0.374)	0.295 (0.237–0.353)	0.951 (0.903–0.998)
2+	0.730 (0.629–0.831)	0.619 (0.558–0.680)	0.367 (0.289–0.445)	0.883 (0.835–0.931)
3+	0.500 (0.386–0.614)	0.828 (0.781–0.875)	0.468 (0.358–0.578)	0.845 (0.799–0.891)
4+	0.284 (0.181–0.387)	0.906 (0.869–0.942)	0.477 (0.330–0.625)	0.807 (0.760–0.853)
5+	0.081 (0.019–0.143)	0.988 (0.974–1.000)	0.667 (0.359–0.975)	0.780 (0.734–0.826)

## Discussion

High risk for ASD was predicted by five contributing items, namely narrowly restricted range of interest, excessive preoccupation with activity or routine, lack of social/emotional conventions when socializing, excessive or unusual reaction to sensory stimuli, and difficulty adapting to even minor change. The contributing items are all well-known signs and symptoms of autism (1). Moreover, several of these items represent some of the earliest behavioral symptoms in ASD. For example, studies have found that some of the signs that are often noticed and reported first by parents include repetitive interests and behaviors, atypical social emotional responses, and extremes of behavioral activity (4, 48, 49). Furthermore, several studies that examined and coded family home videos found differences in repetitive behaviors, social behaviors, and sensory oriented behaviors between those with ASD and typically developing children; these differences were detectable as early as 12 months-old (50, 51). Therefore, in addition to the contributing items of the ASSC scale representing many of the typical symptoms of ASD, research has found that they are also some of the most commonly reported initial concerns.

### Use and Utility of ASSC

Based on the findings, ASSC provides an empirically based score that may be used to identify toddlers, children, and youth who present with signs and symptoms that are known to increase one's likelihood of being diagnosed with ASD. Findings indicate that the ASSC is a good predictor of autism and has reasonable sensitivity and specificity at the designated cut-point of 2+. As a result, it will allow service providers to make more systematic evaluations in determining whether an individual is at greater risk of having ASD, which ultimately helps facilitate prioritization and triaging.

While the ASSC scale has reasonable sensitivity (0.73) and specificity (0.62) at the recommended cut-point of 2+, its PPV at this level is 0.367. This means that ~37% of children and adolescents above the designated cut-point will likely be diagnosed with autism. Although this percentage seems somewhat low, there are a couple of important considerations to take into account. First, both PPV and NPV depend on prevalence, with PPV being directly proportional to the prevalence of autism. This is exemplified in the current study whereby as prevalence of autism increased, for example, from within the trial population dataset to the longitudinal dataset (i.e., 9.5 to 23.3%), the PPV also increased (i.e., from 0.226 to 0.295 for 1+ and to 0.367 for the designated cut-point of 2+). Therefore, utilizing NPV and PPV when prevalence is low should be done with caution, given that one would expect a low PPV. Second, other studies utilizing administrative datasets from real world settings to screen for autism have also reported low PPV (e.g., 0.11) on previously validated instruments (52–55).

Service providers who have completed the interRAI ChYMH, ChYMH-DD, or interRAI Early Years assessment can obtain the ASSC results automatically from the software in which the scale is embedded. It is important to emphasize that the results are meant to assist healthcare providers in identifying how to best support each child's care planning needs based on the ASSC score. Thus, the scale is not meant to be used as an automated decision-making system, without any clinical judgment, but in conjunction with all of the other information collected during the assessment process. Lastly, it is important to always consult with the individual child and the family to ensure that their strengths and needs are considered throughout the process.

Subsequent assessment and care planning steps will be determined, in part, by whether the child's ASSC score falls within the upper or lower range. For example, if the child's score falls within the lower range, it is advised that the healthcare clinicians discuss whether the ASSC score is fitting in light of all of the information that has been gathered. However, if the child's score falls within the upper range, it is advised that the healthcare clinicians consider the individual to be at higher risk for having autism and conduct an in-depth evaluation specifically designed for this sub-population. Ultimately, the key advantage of implementing the ASSC scale would be that toddlers, children, and youth with higher levels of risk should be receiving a timelier comprehensive follow-up evaluation compared to those with lower-level risk. Notably, future research will be conducted to assign ASSC scores to ascending risk categories to determine what labels are best utilized for specific scores on the scale (e.g., highly probable), as well as how they relate to clinical referrals and specific actions that are recommended.

It is important to note that diagnosing autism is a complex process and requires multiple steps, such as: (a) reviewing records; (b) interviewing parents, family members, and other caregivers; (c) assessing for core features through interactions with the child to examine social interaction and communication abilities; (d) utilizing ASD-specific diagnostic tools; and (e) conducting a physical examination and additional investigations (20). Given that the ASSC scale is a brief 5-item measure of key signs and symptoms of autism, it should only be used as an initial screening tool as part of routine practice within a population-based sample. In jurisdictions where the ChYMH or ChYMH-DD is done routinely, the ASSC scale is available at no added cost to provide an additional point of evidence that the team can weigh in the decision-making process. Best practice for a diagnosis of autism is often conducted within a team-based approach utilizing a multi-modal assessment process. In many Canadian jurisdictions, an inter-disciplinary or multidisciplinary specialized team comprised of various health care practitioners work collaboratively in an integrated and coordinated fashion to establish an ASD diagnosis (56), as well as consider differential diagnoses and co-occurring conditions (20).

While the ASSC scale provides an initial screening as opposed to a diagnostic tool, it still has significant utility for individuals, families, clinicians, and the system as a whole. For example, this effective screening method could reduce wait times, allowing for more efficient referrals, and thus quicker access to health, social support, and education services. Notably, earlier access to intervention can foster appropriate development in social interactions, communication, and behavior (57). Therefore, improving access to early diagnosis for young children will capitalize on key developmental windows, increasing the effectiveness of interventions, thereby enhancing prognostic outcomes (8).

Families will also benefit from implementation of the ASSC; more specifically, utilizing an existing instrument that is already in use across most mental health agencies can facilitate expedited triaging resulting in a reduction in the waiting period for families. Such an approach would not only reduce the stress level of the family when navigating the service system, but also foster an increased likelihood of more appropriate referrals to specialized autism services. Research has shown that most families experience the “diagnostic odyssey” as overwhelmingly negative due to long waiting periods. For example, Lappé et al. (58) found that these lengthy wait times caused parents to experience feelings of frustration and a profound sense of uncertainty. Each obstacle and delay within the diagnostic journey has the potential to erode the trust the family has in the healthcare system and their willingness to interact with it (59). Furthermore, as parents continue to wait for an assessment and their stress levels increase, they may be more likely to seek alternative non-evidence-based treatments for their child (60).

With respect to clinical utility, the ASSC provides an opportunity for initial screening for children and youth who are referred for mental health services. Consequently, children who are at greater risk of having autism can be identified through this initial screening approach, resulting in reduced cost and time on behalf of the clinician, agency, as well as the client and their family. Additionally, if a child is determined to be at high risk of having autism and is eventually referred for a more comprehensive diagnostic assessment, a substantial amount of background information will already be available from the interRAI assessment, thereby reducing assessor burden. Interestingly, one study examined factors influencing wait times for an ASD diagnosis and found that the most important predictor of assessment duration was the amount of information available in relation to the child prior to the assessment (61), exemplifying positive downstream effects of the interRAI assessment-to-intervention approach. Thus, increased efficiency will improve early identification, prioritization, and triaging, which will improve the referral-assessment-diagnostic and care pathway as a whole.

At the systems level, comprehensive assessment of ASD is associated with more healthcare costs and resources. Proper screening and triaging utilizing the ASSC can aid in more expedient and efficient use of resources. Having said that, use and utility may differ depending on the resources available as well as the challenges of operationalizing services that are more or less precious. For example, trading off wait times for follow-up diagnosis may look different if a child is seeking services in an urban center in comparison to a remote area or developing nation.

In addition to individualized care planning, the ASSC scale can also provide comprehensive, standardized data across large catchment areas, which: (1) enhances early identification of children with possible autism across the system and (2) provides the ability to examine the prevalence of these symptoms across jurisdictions. Furthermore, this streamlined screening method may help decrease disparities in access to diagnostic services, providing more equitable care at the population-level (62). In addition to the potential impact the ASSC scale can have on patient outcomes, it can also be more cost-effective to the healthcare system as a whole (8, 63, 64). Therefore, our initial screening tool has the potential to make a meaningful impact at both the individual and societal level.

While the current study has many notable strengths, it also has some limitations. For example, given that the present study was not conducted within the controlled setting of a rigorous research study, reliance on broad signals related to associations between items and an autism diagnosis is required, thereby limiting psychometric precision. As such, these results suggest that augmented assessment approaches are needed to reduce disparities to enhance early detection (53). To improve screening and diagnostic precision, new and innovative approaches could be developed that integrate the ASSC with video models (65), new technological advancements, as well as machine learning (52, 66, 67).

Another limitation is that the results of the study may not be generalizable to a community-based sample. This is due to the fact that the young persons assessed were receiving services from inpatient and outpatient mental health agencies. Therefore, future research will explore whether the results of the current study are similar when the young persons assessed are comprised of a community sample. Furthermore, future research will also examine the scale's utility at different cut points once further implementation of the instruments are done both nationally and internationally.

## Conclusion and Implications

There is an overwhelming call for an evolution of the systems of care built to identify those with ASD, as the current wait time to receive a diagnosis is unacceptable to the individuals and families we serve (20, 22, 68). This critical need necessitates the development of an easily accessible and effective screening method. The ASSC scale provides an initial screen for larger populations being assessed as part of routine practice to help identify children and youth at heightened risk for autism. Overall, the main goal for the development and implementation of the ASSC scale is to harness the power of the existing interRAI assessment system to provide a more streamlined screening and referral process. This approach to screening can contribute to earlier identification and intervention, ultimately leading to better patient outcomes at the individual level, and more effective care pathways at the systems level.

## Data Availability Statement

The original contributions presented in the study are included in the article/Supplementary Material, further inquiries can be directed to the corresponding author/s.

## Ethics Statement

The studies involving human participants were reviewed and approved by Western University's ethics review board (REB #106415). Written informed consent from the participants' legal guardian/next of kin was not required to participate in this study in accordance with the national legislation and the institutional requirements.

## Author Contributions

SS and JP developed the analytical strategy. JP performed the statistical analysis. All authors contributed to the formulation of the ideas presented in the study, provided critical feedback to the manuscript, and were involved in the writing and reviewing of the final manuscript.

## Conflict of Interest

The authors declare that the research was conducted in the absence of any commercial or financial relationships that could be construed as a potential conflict of interest. The handling editor JF declared a past co-authorship/collaboration with one of the authors SS.

## Publisher's Note

All claims expressed in this article are solely those of the authors and do not necessarily represent those of their affiliated organizations, or those of the publisher, the editors and the reviewers. Any product that may be evaluated in this article, or claim that may be made by its manufacturer, is not guaranteed or endorsed by the publisher.
